# Structured Syncope Care Pathways Based on Lean Six Sigma Methodology Optimises Resource Use with Shorter Time to Diagnosis and Increased Diagnostic Yield

**DOI:** 10.1371/journal.pone.0100208

**Published:** 2014-06-13

**Authors:** Leon Martens, Grahame Goode, Johan F. H. Wold, Lionel Beck, Georgina Martin, Christian Perings, Pelle Stolt, Lucas Baggerman

**Affiliations:** 1 Medtronic Hospital Solutions, Heerlen, The Netherlands; 2 Blackpool Victoria Hospital, Blackpool, United Kingdom; 3 Medical Center Alkmaar, Alkmaar, The Netherlands; 4 Centre Hospitalier Universitaire Carémeau, Nîmes, France; 5 Northern General/Royal Hallamshire Hospital, Sheffield, United Kingdom; 6 St. Marien Hospital, Lünen, Germany; 7 MagliaRotta, Basel, Switzerland; University of Maryland, School of Medicine, United States of America

## Abstract

**Aims:**

To conduct a pilot study on the potential to optimise care pathways in syncope/Transient Loss of Consciousness management by using Lean Six Sigma methodology while maintaining compliance with ESC and/or NICE guidelines.

**Methods:**

Five hospitals in four European countries took part. The Lean Six Sigma methodology consisted of 3 phases: 1) Assessment phase, in which baseline performance was mapped in each centre, processes were evaluated and a new operational model was developed with an improvement plan that included best practices and change management; 2) Improvement phase, in which optimisation pathways and standardised best practice tools and forms were developed and implemented. Staff were trained on new processes and change-management support provided; 3) Sustaining phase, which included support, refinement of tools and metrics. The impact of the implementation of new pathways was evaluated on number of tests performed, diagnostic yield, time to diagnosis and compliance with guidelines. One hospital with focus on geriatric populations was analysed separately from the other four.

**Results:**

With the new pathways, there was a 59% reduction in the average time to diagnosis (p = 0.048) and a 75% increase in diagnostic yield (p = 0.007). There was a marked reduction in repetitions of diagnostic tests and improved prioritisation of indicated tests.

**Conclusions:**

Applying a structured Lean Six Sigma based methodology to pathways for syncope management has the potential to improve time to diagnosis and diagnostic yield.

## Background

Syncope and transient loss of consciousness (TLoC) account for a substantial proportion of healthcare resource use. Data from the United Kingdom (UK) show that in 2010–11, syncope and collapse were among the top three cardiology-related admissions and that >90% of syncopal admissions are to emergency departments (ED) [1]. In Germany, around 150,000 syncope-related hospitalisations are reported yearly.

Syncope presents a diagnostic conundrum to clinics. Patients are usually recovered by the time of presentation and the event itself was rarely captured by qualified bystanders. As a consequence, a large number of tests are typically employed to detect underlying aetiologies and the patient journey from presentation to diagnosis is often unstructured [2] with low diagnostic yields, often <50% [3].

An analysis from the multicentre PICTURE registry of syncope care in Europe [4] reported an average of 3 different specialists evaluating the typical patient and a median of 13 diagnostic tests performed, including many non-essential and expensive evaluations such as MRI/CT scan, neurological or psychiatric evaluation in almost 50% of patients. Other studies have reported rates of 40% [5]. This long and resource-intense time from presentation to diagnosis results in costs and in lengthy delays in delivery of the appropriate treatment. In some patients, notably those with cardiac syncope, such delays mean the patient may remain at increased risk for cardiac events over substantial time periods instead of receiving appropriate therapies [6], [7].

An unstructured diagnostic strategy, while adhering to guidelines and satisfying clinical necessities, can lead to major insufficiencies in the system. The use of standardised protocols improves diagnostic yields and reduces hospital stay and in consequence, the establishment of syncope units in hospitals has been strongly recommended [8]. A clinical care pathway defines goals and key elements based on guidelines, best practice and patient expectations; it sets the sequence and co-ordinates activities of the multidisciplinary care team, patients and their relatives [9].

A hurdle is that the analysis and implementation of structural changes is often outside the clinical core competence of hospitals. We here describe the preliminary results of as pilot study on the application of principles from process improvement in commercial businesses, with a focus on quality control and lean management, to care pathways for syncope management in hospitals. Lean Six Sigma methodology [10] was used to analyse and evaluate current patient journeys and to design and implement improved care pathways in close collaboration between the hospital teams and the Lean Six Sigma specialists. The project was designed to be compliant with current guidelines and to be process-focused, with no interference with clinicians' expertise, judgement and treatment of patients.

## Methods

Four hospitals took part in the project: The Northern General/Royal Hallamshire Hospital Sheffield and Victoria Hospital Blackpool in the UK, St. Marien Hospital Lünen, Germany, and CHU de Nîmes, France. The hospitals were included in the study based on a willingness to change and try out alternative approaches to their management pathways. There were up to four-fold variations in the sizes of hospitals, catchment areas and annual budgets between the participating centres (Table 1) but none of them specialised in syncope. In addition, the methodological approach was implemented at the Medical Center Alkmaar in The Netherlands. This hospital has a large geriatric patient population, for which additional tests (e.g. cognitive tests and nutrient tests) and a comprehensive geriatric assessment were performed. This made it an outlier and the data were analysed separately.

**Table 1:** Characteristics of the hospitals taking part in the pathway improvement project.

Hospital	Northern General & Royal Hallamshire	Victoria Hospital	St. Marien	CHU de Nîmes	MCA Alkmaar
Catchment area (population ×10^3^)	2000	440	Not Reported	∼700	∼300
Number of beds	∼1100 (Northern General) and ∼850 (Royal Hallamshire) respectively	767	608*	1482	724
Approximate number of syncope patients seen per year	Not Reported	730 (of which 630 on an emergency basis†	Not Reported	N/A	∼700
Approximate annual turnover related to Patient Healthcare Provision	£717 million‡ (Sheffield Teaching Hospitals NHS Foundation Trust)	£256.1 million§ (Sheffield Teaching Hospitals NHS Foundation Trust)	Not separately reported.	€312.3 million**	€251 million††

*Source: http://www.klinikum-luenen.de.

† Hospital Episode Statistics 2006/2007. Source: http://www.hesonline.nhs.uk/HES2/jsp/query_diag.jsp.

‡ Source: http://www.sth.nhs.uk/clientfiles/File/Final%20nhs%20report%20and%20accounts%202012.pdf.

§ Source: http://www.bfwhospitals.nhs.uk/departments/comms/docs/publications/annual_reports/Annual%20Report%202011_12.pdf.

** Source: http://www.chu-nimes.fr/docs/institutionnel/chiffres_cles_2011.pdf.

†† Source: http://www.mca.nl/Portals/6/Bijlagen/Over%20ons/Over%20MCA/Organisatie/Jaarverslag/jaardocument%20en%20jaarrekening%202011%20MCA.pdf.

The patients included in the analyses were selected randomly from those presenting at the hospital with syncope of undiagnosed cause. However, the nature of the study precluded a formal randomisation of patients to the pre- and post- intervention groups.

Multidisciplinary project teams were formed at each centre with members representing a variety of specialties: Lean Six Sigma experts from the sponsor, consultant physicians, nurses, technicians and planners. The project studied processes, not treatments or patients; nor did the team evaluate or interfere with physicians' clinical judgement and treatments. Hence, no approvals were necessary by the hospital ethical review boards. However, the analysis phase sometimes required access to retrospective patient records to chart their journey through the system. Hence, all participating centres and consultants signed confidentiality agreements including clauses safeguarding patient privacy. All patient records/information was anonymised and de-identified prior to analysis.

The objective was to use Lean Six Sigma methodology to identify scope for efficiency gains and to design and implement improved care pathways for patients presenting with recent syncope/TLoC. The analysis focused on two main improvements: the reduction of non-essential tests in a standardisation of steps in diagnostic pathways and a reduction in the time to patient diagnosis. Physicians were cardiologists and neurologists with special interest in syncope and willingness to improve cross speciality interactions in their hospitals. There was no interference with physicians' clinical judgements.

**Figure 1:**
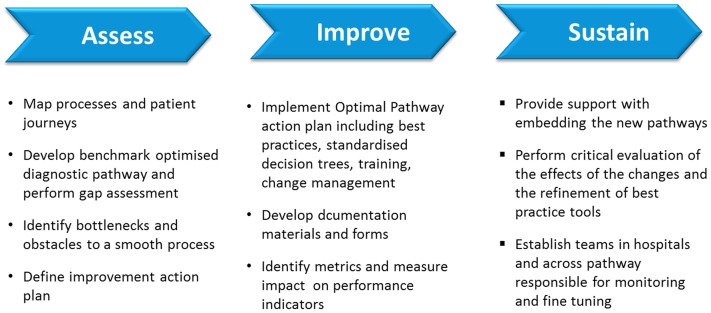
Schematic representation of the Lean Six Sigma methodology used in the care pathway improvent project.

Lean Six Sigma is a highly structured methodology [10] that uses a set of management and statistical methods to optimise business processes. Initiatives are planned and implemented on a project-by-project basis. The typical sequence consists of five steps: Define, Measure, Analyse, Improve and Control (DMAIC). For the current application, these steps were condensed into 3 specific phases, each relying on the previous one for success (Figure 1). Each step had specific, quantified targets.

The first, assessment phase, was mainly driven by the Lean Six Sigma experts who conducted a detailed analysis of baseline conditions at the participating hospitals. Performance was mapped at each centre during a time period of 1–3 months during with patients were followed in their pathways through the diagnostic processes. Assessments included detailed observations of activities, use of time and the resources drawn upon at each step of the pathway. A consolidated map of patients' journeys through the system from admission to diagnosis or discharge was produced in a standardised format. The charts provided a graphic overview of the similarities and disparities in pathways between different patients for further evaluation.

Optimised diagnostic pathways for the average syncope patient were generated based on guidelines for syncope management: NICE guidelines [11] for the UK hospitals; ESC guidelines [7] for the hospitals in continental Europe. A gap analysis was performed on the current processes and patient flows against the benchmark pathways to identify bottlenecks and obstacles to a smooth process. A report with the analysis and key points for improvement was presented to the interdisciplinary team and used to start the second, improvement phase.

The improvement phase was a collaborative effort between all specialties represented on the teams, and lasted for 3–6 months. Optimisation pathways and standardised best practice tools were developed. A new operational model was designed and an “optimal care pathway action plan” was drafted that included best practices and standardised decision trees. Decisions on prioritisation of new measures and processes were taken by the team after considerations of the impact on employees and ease of implementation. Documentation materials and forms were developed around the new pathways to ensure consistent implementation. Specific metrics were identified to measure the performance and the effects of new processes.

To smooth the implementation of the new processes, the action plan included specific staff-training activities on the new processes, as well as support with change-management activities and counselling.

The final, sustain phase, included continuing support with implementing the new pathways, a critical evaluation of the effects of the changes and the refinement of best practice tools. Specific multidisciplinary teams were established in the hospitals, with responsibility for the monitoring and fine tuning of new processes.

The results of the new care pathways were assessed 3–9 months after the end of the improvement phase, at the point in time where the implemented changes were judged by the implementation teams as stable and sustained. A number of key indications were specified. Hospital resources were calculated as the overall cost per patient treated; number of tests taken; time from presentation to diagnosis and the time spent on each patient by physician, nurse or technician. Patient outcomes were evaluated on the diagnostic yield, which was defined as a diagnosis of the cause of syncope or a conclusion leading to a treatment. The patient experience was captured in the overall duration of patient journeys and diagnostic yield. Compliance with guidelines was monitored.

A cost analysis is currently in progress that will include costs for time to diagnosis and associated hospital stay. The results of this analysis will be published separately.

## Results

The different pilot-projects were carried out between 2007 and 2012 over an average time period of 1 year (min. 8 months – max. 14 months). The average size of the project teams in the participating hospitals was 4 individuals (1 doctor, 2 nurses, 1 consultant). Some additional resources were drawn upon as necessary such as finance and IT departments.

### Assessment phase

**Figure 2:**
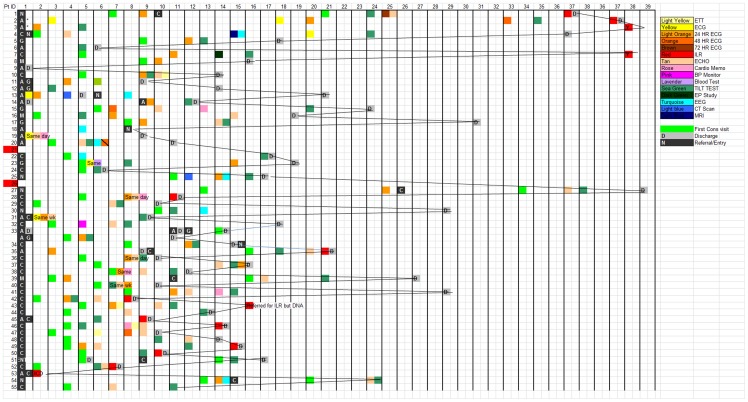
Example of summary chart of patients' journeys in a single hospital. The horizontal axis represents time; patient entry is marked with the black box on the far right. Each coloured square is a visit or diagnostic test. A list of the possible diagnostic tests is shown in the legend on upper right. Shaded squares represent discharge. As can be seen, the sequence of each patient journey is different and the waiting times are long and vary greatly between patients.

As illustrated by a typical chart of patients' journeys in one representative centre (Figure 2), care pathways at the beginning of the project were highly unstructured and there was little consistency in the number and kinds of tests performed, or in process durations between different patients. Although the diagnostic pathways were found to be compliant with current guidelines for the diagnosis and management of syncope, the inconsistencies and differences between centres and patients showed significant scope for improvements. The kinds of tests used in the centres are shown in Figure 2.

### Overall results

The assessment of the overall results were based on a total of 455 patients, 223 in the assessment phase and 232 admitted after the implementation of the new processes. Data on the number of patients in the post-intervention phase were lacking from Northern General & Royal Hallamshire. Only 20 patients were included at the CHU de Nîmes. Hence, p values could not be calculated for all data from these centres.

The new processes led to statistically significant, sometimes dramatic improvements in the performance indicators at all participating hospitals (Table 2). Overall the new processes led to a more efficient use of diagnostic tests and an improved prioritisation of the nature and order of tests. The number of tests was reduced by on average 24%; p = 0.046. Median time from admission to diagnosis was shortened by on average 59% (p<0.048). The efficiency improvements came together with an increased diagnostic yield: from 42% before the implementation of new processes to 73% with the new systems (p = 0.007). All use of the assessed newly implemented processes (100%) was compliant with guidelines.

**Table 2:** Mean number of diagnostic tests, diagnostic yields and median times to diagnosis in the overall study group and at the individual participating hospitals before and after implementation of new pathways.

		All centres*	Northern General & Royal Hallamshire	Victoria Hospital	St. Marien	CHU de Nîmes	MCA Alkmaar†
N	Pre	223	89	55	41	12	26
	Post	232	Not reported	58	51	8	115
Average number of tests	Pre	4.125	4.5	4.0	4.0	4.0	2.4
	Post	3.15	4.0	2.2	3.4	3.0	7.0
	Change	−24%	−11%	−45%	−15%	−25%	+192%
	p value‡	<0.046	Not reported	<0.001	0.041	Not reported	NA
Diagnostic yield	Pre	42%	Not reported	46%	37%	Not reported	81%
	Post	73%	Not reported	83%	62%	Not reported	95%
	Change	+75%	NA	+80%	+68%	NA	+17%
	p value§	0.007	NA	<0.001	0.013	NA	0.016
Median time from admission to diagnosis (days)	Pre	21	41	17.5	7	30	13
	Post	9	14	1	7	15	7
	Change	−59%	−66%	−99%	0%	−50%	−46%
	p value**	<0.048	Not reported	<0.001	NA	Not reported	<0.001

* Excluding MCA Alkmaar.

† Specialist geriatric centre.

‡ Paired T test.

§ Chi-Square test.

** Mood Median test.

The implantation of an insertable loop recorder (ILR) is often relevant to hospitals, as this decision is based on a well-founded suspicion of cardiac causes of syncope obtained by other tests. ILRs provide data over several years of follow-up and thus for a hospital, an ILR implant represents discharge and the end of a patient journey. When the definition of diagnostic yield was expanded to include ILR implant, yields were highly similar to those without including the ILR implant decision: 60% before the implementation of the procedures and 86% with the new procedures in place.

### MCA Alkmaar

The MCA Alkmaar was unique in having a large geriatric population. The number of tests increased at this centre with the new processes as more tests such as nutritional status were introduced to improve diagnostic yield. Despite the increased number of tests, the time to diagnosis was shortened significantly at MCA Alkmaar, to similar extent as in the other centres (Table 2).

### Case study: Victoria Hospital Blackpool

As the individual hospitals varied in their needs and in the nature of the specific changes implemented to their processes, we describe the experiences in one specific centre, Victoria Hospital in Blackpool (UK). The characteristics of this hospital are shown in Table 1 and Table 2. At baseline, the median time per patient from presentation at the hospital to diagnosis was 13.5 weeks and varied widely, up to 39 weeks.

The objectives of the project were defined at the outset: the lead time from presentation to treatment should not exceed 18 weeks and the proportion of patients recommencing the diagnosis-to-treatment loop should be reduced to <5%. In addition, unnecessary admissions of low to medium risk patients to the accident & emergency (A&E) department should be reduced. It was imperative to maintain compliance with NICE guidelines. The project team had 11 members, including managers, nurses and consultants from cardiology, ED and neurology.

The assessment phase identified several points of potential efficiency increases. As there was no multidisciplinary approach to patient management, standardised referral criteria, definitions of blackout and standardised pathways were all missing and the content and quality of patient histories varied widely. Patients came into the diagnostic pathways at a multiplicity of entry points. Around half of patients presented after referrals from general practitioners and many of these referrals were of low quality or inappropriate. In consequence, a large proportion of patients (37%) were caught in a loop of repeated diagnosis, which slowed their progress through the pathway. A third of patients were referred by cardiologists. The remainder presented at the A&E department, were referred by neurologists or by other physicians.

The most important change implemented was the creation of a “one stop shop” – The Lancashire Blackout Clinic – with the aim to provide a diagnosis in all patients admitted with clear referral criteria. The clinic is led by 2 nurses and overseen by the Consultant Cardiologist of the Week. Staff handle patients as they arrive and guide them through the pathways, to ensure a smoother process, clearer focus and optimised patient flow.

The design and implementation of standardised, clear pathways were key to reducing redundancies and waiting times in the patient flow. Clear definitions, rules for risk stratification and timings for the different diagnostic steps were included in the pathway and checks were introduced to ensure these were kept. To reduce the number of inappropriate referrals, clear referral criteria were communicated and embedded in a mandatory referral form for all physicians. The multidisciplinary approach was abolished and replaced by regular interdisciplinary meetings where complex cases are discussed.

**Figure 3:**
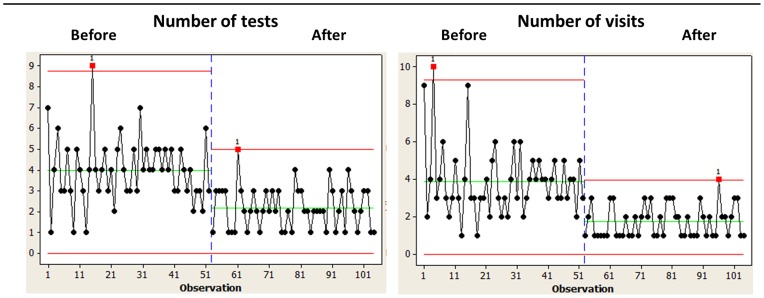
Example of impact of the new diagnostic processes for blackout/syncope at the Victoria Hospital Blackpool. Number of tests (left-hand graph) and visits (right-hand graph) are shown before and after implementation of the new processes.

**Figure 4:**
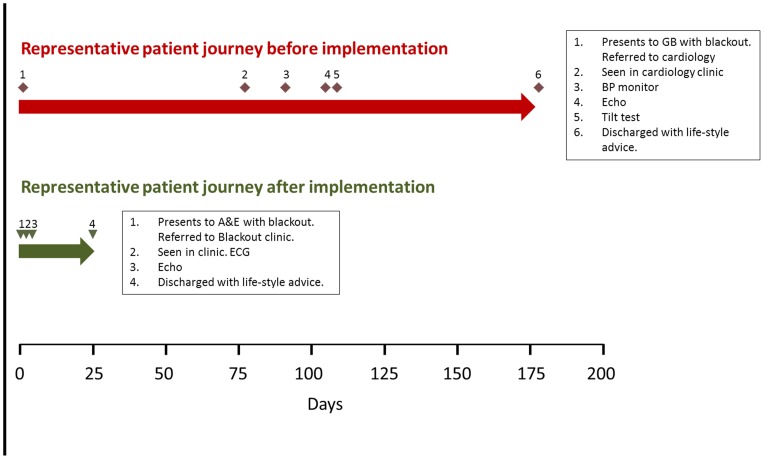
Two representative examples of patient journeys before and after implementation of Lean Six Sigma derived syncope pathways at Victoria Hospital Blackpool. Red diamonds indicate points on the patient journey before the implementation of new pathways; green triangles show points on the journey with the new pathways structured after the Lean SIx Sigma project. Events in the patient journey and performed tests are noted in the boxes.

As a result of the changes, the overall median lead time from referral to diagnosis was reduced from 161 days to 54 days, that from referral to first appointment from 20.5 to 14.2 days and the time from first visit to diagnosis from 17 to 1 day. The volume of referrals coming from outside the hospital's catchment area was reduced significantly. On average, 2.2 test were conducted with the new procedures v 4.0 in the old system, and the average number of visits per patient was reduced from 4 to 1.75 (Figure 3). Table 3 lists the test performed before and after project implementation. A comparison of representative patient journeys before and after implementation of the new syncope care pathways, including the tests performed, is shown in Figure 4. The improved pathways were associated with increased diagnostic yield: from 46% to 83% (Table 2).

**Table 3:** Tests performed before and after implementation of the new pathways at the Victoria Hospital Blackpool.

	Per cent of patients with test	
Test	Before	After
Exercise treadmill testing	7%	0%
ECG	13%	100%
24 Hr Holter	55%	15%
48 Hr Holter	7%	0%
72 Hr Holter	2%	0%
Echocardiography	49%	27%
Cardio Memo	11%	10%
BP Monitor	2%	0%
Blood Test	2%	0%
Tilt Test	80%	19%
EP Study	2%	0%
EEG	15%	2%
CT Scan	4%	2%
MRI	2%	0%

## Discussion

The results presented from this pilot study, from four hospitals in three European countries, show a promising potential from applying a structured Six Lean Sigma process-optimisation methodology to the identification of efficiency bottlenecks and to the design and implementation of improved care pathways for patients presenting with syncope/TLoC. Over the 9–15 months assessment, improvement and sustain periods, there were significant improvements in lead times and diagnostic yield. In all hospitals the number of tests was significantly reduced. Even at the centre MCA Alkmaar, with a large geriatric population led to a higher number of relevant tests after the implementation of the new pathways, the new system reduced the time to diagnosis, as well as increasing the diagnostic yield. Compliance with the respective guidelines in each country was maintained.

There is an acknowledged potential for the productive use of Lean or Six Sigma methodologies in health care [12]. To our knowledge, this is the first study to show that a partnership between healthcare providers and experts on process improvement from commercial businesses, with a focus on quality control and lean management, can be used to structure, standardise and optimise care pathways for syncope/TLoC management in hospitals. Other successful attempts to improve syncope management have focused on establishing dedicated syncope units and implementing standardised protocols and sequences of tests [3], [5], [7], [8], [13], [14]. However, there has not previously been a focus on process management and on organising tests in the most effective fashion.

Lean Six Sigma takes a highly structured approach to process improvement [10]. The methodology has two focus areas, efficiency improvement (lean) and improved process quality (six sigma). Our baseline mapping revealed inefficiencies in the processes as well as a high variation in the diagnostic pathways between patients. Both factors were improved by the new pathways. While the Lean Six Sigma methodology is highly structured, there is flexibility within its application. Each situation is analysed individually and the initiatives are planned and implemented according to the needs defined for each specific organisation. This is relevant to hospital environments, which can vary widely in organisation, patient pool and regulation.

One of the most efficient changes was the introduction of standardised referral criteria and mandatory referral forms, as done, e.g., at the Blackpool Victoria Hospital. The standardisation greatly reduced the number of patients sent back because of inadequate referrals or going into a loop of repeated testing. This is a common situation: an observational study in an Irish hospital has reported that 58% of admissions for syncope were unnecessary when compared with ESC guidelines [15]. Even greater improvements than those at the Victoria Hospital may thus be achieved by, e.g., greater use of automatic methods to improve tracking and documentation of patients and visits. Such systems also have the potential to reduce errors due to multiple manual entries of the same data.

In achieving an average of 1 day lead time to diagnosis, Victoria Hospital may be seen as a best case scenario, but improvements were achieved at all centres, indicating that Lean Six Sigma driven processes may be beneficial across a range of local conditions. Even in the case of MCA Alkmaar, where more tests were performed with the new system, the time to diagnosis was reduced. This further shows that the number of tests in itself is not a good measure of quality of care.

Efficiency improvements in healthcare must not come at the costs of reduced quality of care or service to patients. Our results are reassuring on these counts. As untreated cardiac syncope is associated with increased morbidity and mortality [6], a shorter time to diagnosis and appropriate treatment can be expected to reduce mortality. A large part of the improvements can be attributed to reductions in tests not mandated by guidelines. The reasons for the increase in diagnostic yield are probably multifactorial. One factor was the reduction in the number of patients who were sent back because of inadequate referrals, which increased the rate of relevant patients entering the process.

A number of factors can be identified that helped in successful implementation of changes and not all may be transferrable to non-syncope care pathways. The hospitals showed a strong engagement and willingness to commit resources and to implement change management. Without readiness to change, it is very difficult to overcome inertia in any organisation. Also, in the multinational environment of Europe, local knowledge and language skills are necessary to identify and evaluate information specific to each individual hospital.

The allocation of appropriate resources is important at all stages of the process. The assessment phase is labour-intense, but to the hospital the additional personnel resources needed are minor, as most of the work is carried out by the consultants. The changes themselves require dedicated healthcare personnel and structural reorganisation of hospital procedures. The successful outcomes indicate that associated savings would compensate for the associated costs and possibly save overall costs. An analysis of these effects is in preparation. The short time, typically <6 months, until benefits were seen with the new processes increases participant motivation.

The availability of specific guidelines for the diagnosis and management of syncope enabled the generation of optimised diagnostic pathways to use as benchmarks. The complexity of Syncope/TLoC provides greater scope for process optimisation than diagnoses with simple pathways, fewer components and decision points.

Several factors will affect the sustainability of the improvements. Dedicated project owners are necessary and the continuing aid of independent consultants may help. Some of the changes, such as a specially staffed blackout clinic, need resources and dedicated staff which may be susceptible to strategic decisions from hospital management or to political decisions beyond the control of the respective clinics. It is also important to maintain continued support from staff at all levels.

Among weaknesses in this work should be mentioned the lack of formal randomisation of patients. We included patients randomly, but there was no randomised allocation of patients to the procedures before and after the management interventions. As the projects established individual new processes in each hospital it was not possible to run a parallel-group study. There is a theoretical possibility of differences in characteristics between the groups of patients admitted in the pre- and post phases, respectively. The random inclusion of patients, together with the large and consistent benefits with the new processes observed across participating hospitals make it highly unlikely that such systemic differences would exist in the other hospitals.

Other weaknesses are the lack of data on the number of patients in the post-intervention phase at Northern General & Royal Hallamshire and the low number of patients included at the CHU de Nîmes. We did not systematically analyse the kinds of tests performed in the pre- and post- phases, not do we have data on the diagnostic yield of individual tests.

Patient satisfaction was not captured in our analysis. It is known from other studies that diagnostic yield and waiting time are two important factors for patients [16]–[18] (S. Tsintzos, unpublished). Both these factors were reduced with the Lean Six Sigma guided pathways. Recently, the department of Neurosurgery at the UCLA Medical Center applied lean methods to streamline processes and co-ordinating tests, with resulting improvement in patient satisfaction [19].

In summary, adapting a structured Lean Six Sigma approach to process optimisation in a hospital setting improved the efficiency and diagnostic yield of diagnostic pathways for the diagnosis of syncope. With appropriate modifications the strategy should be applicable to a variety of hospital organisations. As syncope represents 5–10% of admissions to an average European hospital and 50%–80% of patients presenting with syncope at an A&E department are admitted for inpatient care [15], [20], [21], wider adaptation of the methods may have beneficial implications on patient outcomes and clinic resources.
